# Caspase-6 Knockout in the 5xFAD Model of Alzheimer’s Disease Reveals Favorable Outcome on Memory and Neurological Hallmarks

**DOI:** 10.3390/ijms21031144

**Published:** 2020-02-09

**Authors:** Ariel Angel, Rotem Volkman, Tabitha Grace Royal, Daniel Offen

**Affiliations:** 1 Laboratory of Neuroscience, Felsenstein Medical Research Center, Sackler Faculty of Medicine, Tel Aviv University, Tel Aviv 6997801, Israel; arielangel1@post.tau.ac.il (A.A.); rotemvol@mail.tau.ac.il (R.V.); 2 Department of Pharmacy, Birla Institute of Technology & Science, Pilani - Hyderabad Campus, Hyderabad 500078, India; f20171174@hyderabad.bits-pilani.ac.in; 3 Sagol School of Neuroscience, Tel-Aviv University, Tel Aviv 6997801, Israel

**Keywords:** Alzheimer’s disease, caspase-6, neurodegeneration, knock-out mice

## Abstract

Alzheimer’s disease (AD) is a progressive neurodegenerative disorder and is the most common form of dementia in the elderly. Caspases, a family of cysteine proteases, are major mediators of apoptosis and inflammation. Caspase-6 is considered to be an up-stream modulator of AD pathogenesis as active caspase-6 is abundant in neuropil threads, neuritic plaques, and neurofibrillary tangles of AD brains. In order to further elucidate the role of caspase-6 activity in the pathogenesis of AD, we produced a double transgenic mouse model, combining the 5xFAD mouse model of AD with caspase-6 knock out (C6-KO) mice. Behavioral examinations of 5xFAD/C6-KO double transgenic mice showed improved performance in spatial learning, memory, and anxiety/risk assessment behavior, as compared to 5xFAD mice. Hippocampal mRNA expression analyses showed significantly reduced levels of inflammatory mediator TNF-α, while the anti-inflammatory cytokine IL-10 was increased in 5xFAD/C6-KO mice. A significant reduction in amyloid-β plaques could be observed and immunohistochemistry analyses showed reduced levels of activated microglia and astrocytes in 5xFAD/C6-KO, compared to 5xFAD mice. Together, these results indicate a substantial role for caspase-6 in the pathology of the 5xFAD model of AD and suggest further validation of caspase-6 as a potential therapeutic target for AD.

## 1. Introduction

Alzheimer’s disease (AD) is an ever-growing neurological disorder, with numbers projected to attain 14 million by 2050 [1]. It is considered one of the leading causes of death in America, and tremendous amounts of money have been spent to combat the disorder to no avail. Progression of the disease is affected by various factors, some of which are well-established and characterized. Amyloid precursor protein (APP) is an internal membrane protein that can be cleaved by several enzymes. The amyloid-β (Aβ) peptide is generated by the proteolytic cleavage of APP by β- and γ-secretases [2]. The β-cleaved peptide can then be aberrantly folded and conform into amyloid oligomers and plaques, which are thought to be an early event in the progression of AD [3]. The APP can also be cleaved at APP/C99 by caspases which, in turn, produces a neurotoxic peptide (C31) [4].

Caspases are a family of proteases that cleave specific target proteins and have a variety of roles in cellular processes. They are divided into two general categories: initiators or executioners. Each caspase can affect inflammation, apoptosis, or both, and they are highly conserved throughout species [5]. Executioner caspases are activated through cleavage of the prodomain, followed by cleavage into smaller subunits that form the active caspase heterodimer [6]. Caspases have been implicated in numerous diseases as their function leads to apoptosis and cell death [7,8]. Elucidating their roles in those diseases has been the subject of many years of research around the world.

Caspase-6 belongs to the pro-apoptotic executioner caspase group and is activated by a series of upstream activators, amongst which are caspase-3 and caspase-1 [9,10]. Caspase-6 was shown to mediate several non-apoptotic processes, such as facilitating axonal pruning during development [11,12]. Cleavage by caspase-6 of the mutant huntingtin gene was shown to be a prerequisite for neuronal dysfunction and degeneration, and therefore, exacerbates neurodegeneration in Huntington’s disease [8,13]. Caspase-6 activity is associated with AD pathological lesions and is present at the early stages of tangle formation [14]. The pro-apoptotic protein p53 is increased in AD brains and p53 directly up-regulates the transcription of caspase-6 [15], resulting in a significant increase in caspase-6 mRNA in human AD brains. Caspase-6 cleaves the APP at a specific site, creating the toxic small peptide C31, a potent inducer of apoptosis in cultured neuroblastoma cells [4]. Thus, caspase-6 is considered to be an up-stream modulator of AD pathogenesis, and as a result, a viable therapeutic target for the treatment of AD [8].

Caspase-6 knock-out (KO) neurons are protected against excitotoxicity, trophic factor deprivation, and myelin induced axonal degeneration [16]. Caspase-6 KO mice were first described by Uribe et al. [16]. Research using these mice showed a reduction in the inflammatory response and behavioral changes following peripheral pro-inflammatory stimuli by lipopolysaccharide (LPS) [17].

Knock-out mice are often used in scientific environments in order to elucidate mechanisms of the targeted proteins. In the present study, we aimed to use the caspase-6 KO mice to better understand its role in AD. The 5-gene familial AD (5xFAD) mice have five distinct human mutations either in the APP or Presenilin-1 (PSEN1) genes [18]. These mice have been used extensively to study AD progression, as well as potential treatments for the diseases, due to their similarity to human AD pathologies.

To this end, we have generated a novel double-KO strain harboring both caspase-6 KO, together with the 5xFAD model. Through behavior tests, protein, and mRNA analysis, we have shown a beneficial effect of caspase-6 KO on memory and inflammatory markers in 5xFAD/C6-KO mice. By evaluating neurological hallmarks, we have shown a reduction in reactive astrocytes and microglia in mice hippocampi, as well as alleviation of plaque burden in 5xFAD/C6-KO mice. This has served us to better evaluate the impact of caspase-6 activity on AD progression and its distinct pathologies.

## 2. Results

### 2.1. Generation of Double Transgenic Mice

In order to evaluate the effects of caspase-6 knock out (KO) in AD, we crossbred 5xFAD [18] mice with caspase-6 KO (-/-) mice [19]. We used double transgenic [5xFAD (+/-) x Caspase-6 (-/-)] 5xFAD/C6-KO mice and compared them to 5xFAD/C6-Het mice [5xFAD (+/-) x Caspase-6 (+/-)], together with their wild type (WT) and KO littermates (Designated WT/C6-Het and WT/C6-KO). Behavior tests started when mice were six and a half months of age.

### 2.2. Caspase-6-KO in 5xFAD Female Mice Improves Behavioral Deficits

**Figure 1:**
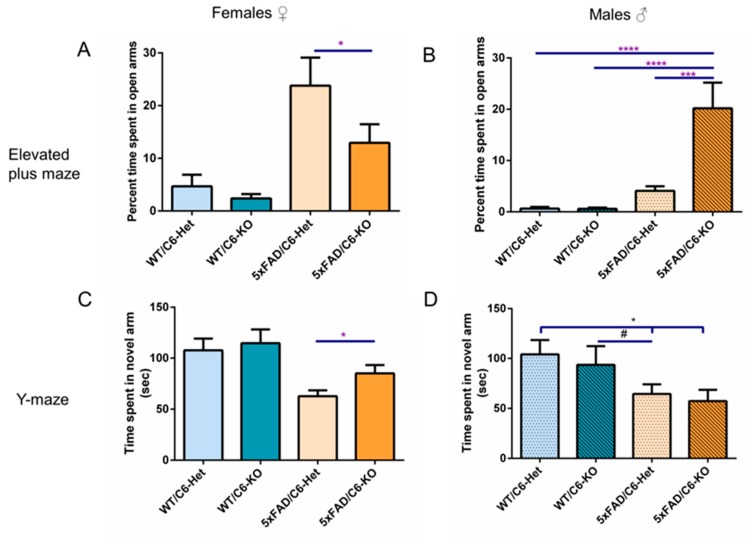
Behavioral amelioration in 5xFAD/C6-KO female mice in the Y-maze and elevated plus maze.

Initially, we examined risk/anxiety-related assessments in the elevated plus maze, where WT mice generally spent more time in the closed arms of the maze, while 5xFAD mice showed tendency toward the open, exposed arms [20]. In our experiment, 5xFAD/C6-Het female mice spent significantly more time in the open arms, reflecting perturbed risk assessment behavior, compared to WT mice. In the female group, 5xFAD/C6-KO mice spent significantly less time in the open arms of the maze compared to 5xFAD/C6-Het mice (Figure 1A). However, male transgenic mice did not appear to follow the expected model (Figure 1B).

Marked improvement is observed in female 5xFAD/C6-KO mice compared to their transgenic counterpart, both in forced alternation Y-maze and Elevated plus maze (Figure 1A,C). Male mice did not show an improvement in the Y-maze (Figure 1D), and results from the elevated plus maze (Figure 1B) are not consistent with the expected 5xFAD model in the plus maze, as transgenic mice did not spend time in the open arms. Data are mean ± SEM. *p< 0.05, #<0.05 ***p < 0.001, ****p < 0.0001. Two-tailed t-test between groups.

Next, we tested whether caspase-6 KO would inhibit the cognitive decline seen in 5xFAD mice. The Y-maze test can be configured to evaluate spatial working memory, which is showed to be impaired at an onset of four to five months of age in 5xFAD mice [18]. Here, 5xFAD/C6-Het female mice showed reduced exploration time in the novel arms as compared to WT mice. Conversely, 5xFAD/C6-KO female mice demonstrated a 30% increase in time spent in the novel arms of the maze as compared to 5xFAD/C6-Het mice (Figure 1C), implying limited cognitive decline among 5xFAD/C6-KO mice. No significant difference in exploration time was measured between 5xFAD/C6-Het and 5xFAD/C6-KO male mice (Figure 1D).

Together, these results indicate that Caspase-6 KO reduces risk assessment, and anxiety and memory-related behavior in female 5xFAD/C6-KO mice.

### 2.3. Caspase-6 KO in 5xFAD Mice Ameliorates Spatial Memory and Learning Time in the Morris Water Maze Test

**Figure 2:**
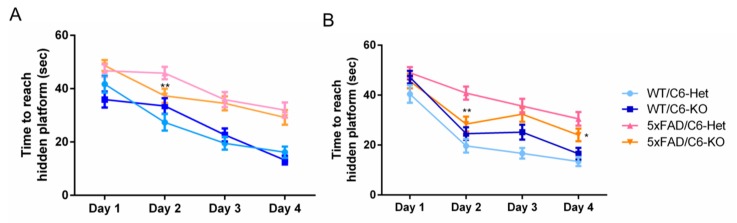
Caspase-6 KO in 5xFAD mice improves cognitive performance in the Morris water maze test.

We then examined spatial learning capacity in these mice in the Morris water maze (MWM) test at seven months of age. During the four days of the trial, both female and male 5xFAD/C6-Het mice showed significantly longer latency to find the platform, reflecting impaired spatial learning (Figure 2A,B respectively). However, both female and male 5xFAD/C6-KO mice showed improved learning ability, demonstrated by a significantly shorter time to find the platform on day two for female mice, and day two and day four in male mice, as compared to 5xFAD/C6-Het mice (Figure 2A,B respectively).

Mice were tested for platform position learning at seven months of age in the MWM test. Mice were trained to find the hidden platform four times a day, for four days. Escape latency for female (Figure 2A) and male (Figure 2B) mice were measured during a 60 s trial. Significant reduction in platform finding time was observed on the second day for 5xFAD/C6-KO male and female mice, and on day four for male mice, as compared to 5xFAD/C6-Het mice. Displayed are averages ± SEM of all four trials per day, per group. *p < 0.05, **p < 0.01. Two-tailed t-test 5xFAD/C6-KO, compared to 5xFAD/C6-Het. Average body weight of animals was identical throughout each group in accordance with gender (Supplementary Figure S1).

**Figure 3:**
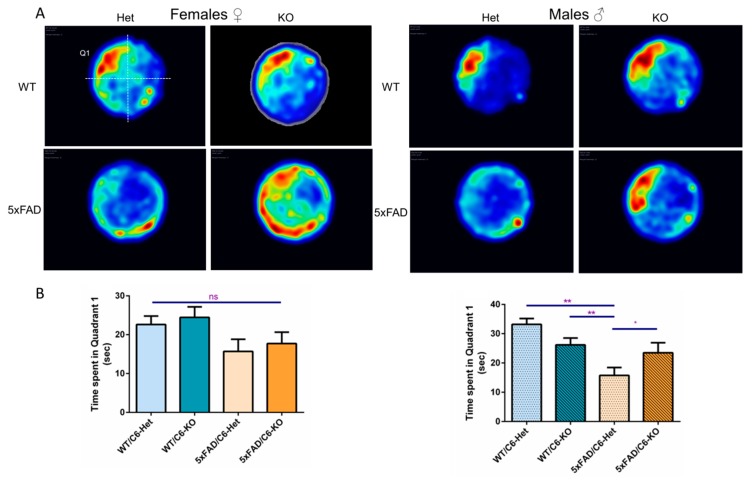
Caspase-6 KO in 5xFAD mice improves searching pattern in the probe test.

The learning improvement in 5xFAD/C6-KO mice could also be reflected in the probe test on day five. While 5xFAD/C6-Het mice exhibited relatively less time in the platform quadrant, 5xFAD/C6-KO male mice showed greater preference and searching behavior around the platform position (Figure 3B). Female 5xFAD/C6-KO mice did not show an improvement compared to their transgenic counterpart (Figure 3B). The probe test data is also illustrated as a heatmap to show the total path and swimming strategy taken by each group. 5xFAD/C6-KO female and male mice show a preference to the platform position and spend most of the probe trial time in quadrant #1 (Q1), where the platform was positioned during the learning phase (Figure 3A).

On the fifth day of the MWM test, the platform was removed, and mice were released from the furthest position to quadrant #1 (Q1).

In Figure 3A: For the heatmaps, mouse movement during the 60 s trial was tracked using Ethovision, taking all animals from each group and combining them in one summarized image. WT-Het mice from female and male groups show a clear preference to the platform position at Q1. Caspase-6 KO female and male mice show no negative effect. 5xFAD/C6-Het female and male mice show no clear path or quadrant preference. In comparison, 5xFAD/C6-KO female and male mice show a strong preference to the platform quadrant, spending most of the 60 s trial in the former platform quadrant or hemisphere. Red and dark blue mark strong or low presence, respectively.

In Figure 3B: Time spent in the former platform position Q1 was evaluated during a 60 s probe trial in female and male mice. Displayed are averages ± SEM. *p < 0.05, **p < 0.01. Two-tailed t-test between marked groups.

### 2.4. Synaptophysin, a Pre-Synaptic Marker, is Up-Regulated in 5xFAD/C6-KO Double Transgenic Mice

**Figure 4:**
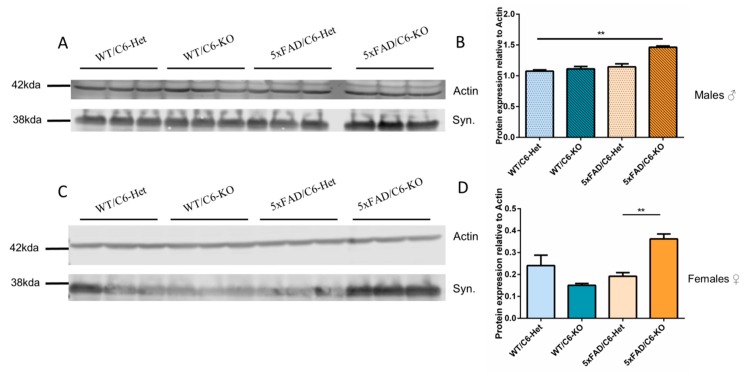
Synaptophysin is increased in 5xFAD/C6-KO double transgenic mice. Syn: Synaptophysin.

During the progression of AD, Aβ causes loss of dendritic spines which subsequently give rise to cognitive dysfunctions [21]. This leads to reduction in potential healthy synapses and strength of connections among neurons. It was also shown that caspase-6 could mediate synapse dysfunction and loss before the occurrence of neurodegeneration and cell death [22]. Synaptophysin, a pre-synaptic marker, is increased dramatically in both male and female 5xFAD/C6-KO mice, suggesting that higher neuronal interaction may occur when caspase-6 is reduced in transgenic mice (Figure 4). This increase in synaptophysin may suggest an improved synaptic density in 5xFAD/C6-KO mice.

Above is a western blot analysis of male (Figure 4A) and female (Figure 4C) hippocampi lysates for synaptophysin. Three mice per group (Average ± SEM) were compared to actin and 30µg loaded protein on 10% SDS gel, and quantified using Licor image studio for male (Figure 4B) and female (Figure 4D) mice. **p < 0.01. Two-tailed t-test between selected groups. Uncropped blots are displayed in supplementary Figure S2.

### 2.5. Gene Expressions are Altered in 5xFAD/C6-KO Double Transgenic Mice

**Figure 5:**
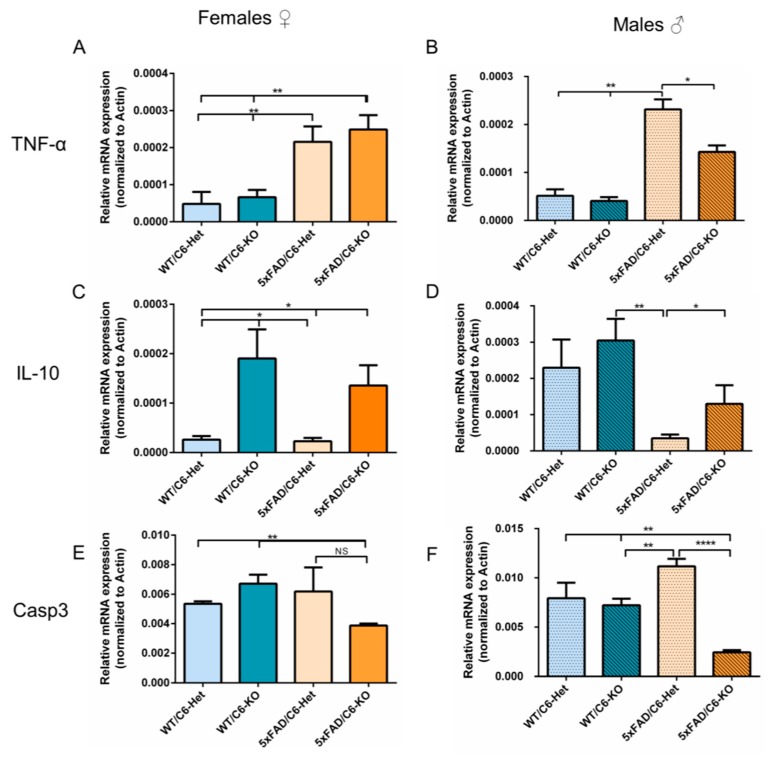
Altered gene expression in hippocampus of 5xFAD/C6-KO mice. Mice hippocampi were taken for RT-PCR analysis of several genes: mRNA quantification for female and male TNF-α (**A**,**B**), IL-10 (**C**,**D**); and Caspase-3 (Casp3; **E**,**F**) respectively. Data are mean ± SEM. *p < 0.05, **p < 0.01, and ****p < 0.0001. Two-tailed t-test between marked groups. NS: Not significant.

Caspase-6 is known to affect inflammation and can be activated by the neuronal Nod-like receptor protein 1 (NRLP1) inflammasome through a caspase-1-dependent pathway, which releases interleukin1-β (IL-1β) and causes caspase-6 mediated axonal degeneration [23]. Pro-inflammatory cytokines, such as tumor necrosis factor alpha (TNF-α), are known to be up-regulated in 5xFAD transgenic mice [24]. In the 5xFAD/C6-KO group, TNF-α levels were significantly reduced in male mice compared to their transgenic counterpart (Figure 5B). Female mice did not display a clear distinction between the two transgenic strains (Figure 5A).

IL-10 is an important negative immune regulator that can prevent damage caused by pro-inflammatory processes, and it was found to be in close relationship with caspase-6 [25]. In both caspase-6 KO groups, IL-10 gene expression was elevated compared to Het groups (Figure 5C,D). This might suggest an up-regulated anti-inflammatory process, which may lead to reduced inflammation in the double transgenic model.

Another gene closely related to caspase-6 is caspase-3, as it was shown that caspase-6 can act as an initiator in the activation of caspase-3 [26]. Here, we show that caspase-3 is reduced in 5xFAD/C6-KO mice compared to 5xFAD/C6-Het male mice (Figure 5F), and with a descending trend in female mice (Figure 5E). A reduction in caspase-3 activity can be directly linked to a reduction in neuronal cell death, as well as plaque formation [27].

Additional pro-inflammatory cytokines, IL-6 and IL-1β, are known to increase in AD and play a fundamental role in the progression of the disease [28,29]. Gene expression of both these genes in male and female 5xFAD/C6-KO mice were not altered, compared to their 5xFAD/C6-Het counterpart [Supplementary Figure S3]. Although caspase-6 plays a role in the increase of these cytokines [17,23], they can still be up-regulated by many other factors while caspase-6 is absent [30,31].

### 2.6. Amyloid-β Aggregates in the Hippocampus are Reduced in Caspase-6 KO Double Transgenic Mice

**Figure 6:**
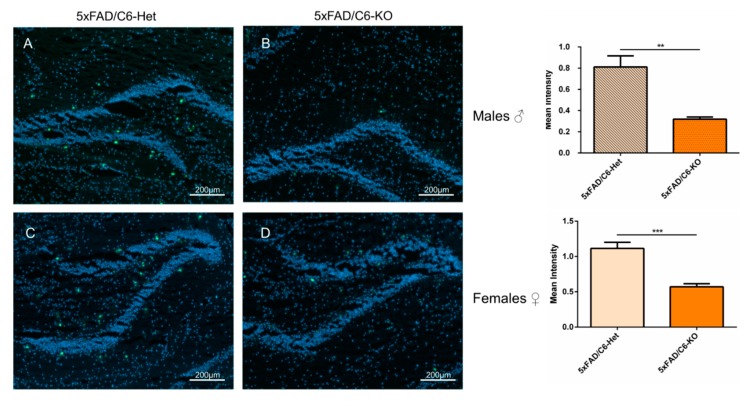
Caspase-6 KO in 5xFAD mice reduces amyloid-β plaque load. Representative images of hippocampal slices stained with ThioS for male (**A**–**B**) and female (**C**–**D**) transgenic mice and their respective quantification (*n* = 5–7). Scale bar = 200μm. Data are mean ± SEM. **p< 0.01 and ***p < 0.001. Two-tailed t-test.

Aβ aggregation is one of the main pathologies of AD, and in the 5xFAD mouse model, extracellular amyloid deposition begins at two months of age [18]. Thioflavin S (ThioS) staining is often used as a marker of fibrillar material with a β-pleated sheet conformation [32]. This staining method can quite reliably detect intraneuronal Aβ42 in its aggregated stage in this AD model. 5xFAD/C6-Het mice show extensive depositions and aggregates of plaques in the dentate gyrus area of the hippocampus. When comparing the two transgenic groups, a significant reduction in plaque depositions is detected in 5xFAD/C6-KO mice (Figure 6). This finding can support the positive effect in 5xFAD/C6-KO mice on memory task performance as plaque burden is associated with hippocampal memory [33,34].

### 2.7. Activated Astrocyte and Microglia are Reduced in 5xFAD/C6-KO Mice

**Figure 7:**
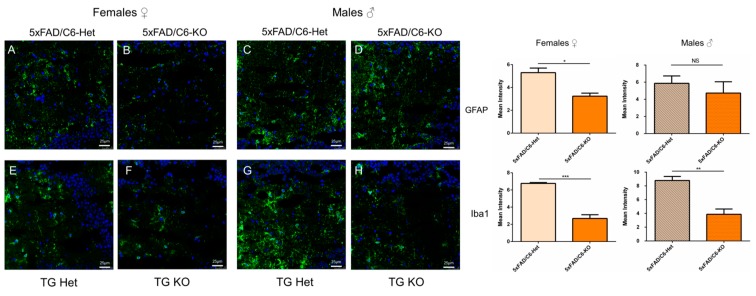
Low IBA1 and GFAP expression in 5xFAD caspase-6 KO mice. Representative images of hippocampal slices stained with GFAP (**A**–**D**) or IBA1 (**E**–**H**). Quantification of GFAP and IBA1 are mean intensity of *n* = Scale bar = 25 μm. Data are mean ±SEM. *p < 0.05, **p < 0.01, and ***p < 0.001. Two-tailed t-test.

To further investigate the neurological impact of caspase-6 KO mice, markers of astrocytes and microglia were analyzed. Glial fibrillary acidic protein (GFAP) is involved in controlling the shape, movement, and function of astroglial cells. Increased GFAP immunoreactivity is considered to represent an index of gliosis and gradually developing neural damage [35], and it is known to increase in the 5xFAD model of AD [36,37]. Previous work has shown that overexpression of caspase-6 in the mouse hippocampus increases astrocyte and microglial activation, essential signs of inflammation in the CNS [38]. Here, we show that GFAP is reduced in 5xFAD/C6-KO female mice compared to 5xFAD/C6-Het transgenic mice, but not in male mice (Figure 7A–D). Additionally, the ionized calcium binding adaptor molecule 1 (IBA1), a marker of activated microglia [39], is reduced in both male and female double transgenic mice (Figure 7E–H). These results demonstrate further the effects of caspase-6 activity, or lack-thereof, on a broad spectrum of cells and processes.

## 3. Discussion

Caspase-6 inhibition has been a scientific goal for many years in various fields and research labs. This is due to its broad effect in a myriad of pathologies, from cancer malignancies to neurological disorders, as well as pain and inflammation.

In order to attain a clear picture of the effects of caspase-6 KO on 5xFAD mice, both males and females were tested separately. Many studies have shown and emphasized the importance of sex differences in animal studies. Social, anxiety, memory, and locomotor behaviors were all affected by the sex of the animal [40,41]. Indeed, in the 5xFAD mouse model, a drastic phenotype is observed in aging females, leading to decreased motor behavior and sometimes paralysis and death [42]. In our study, several female mice were humanely euthanized prior to behavior testing, due to weight loss and impaired movement of lower limbs. Seizures were also observed in several 5xFAD female mice in both Het and KO genotypes [43]. Male mice did not display any seizure or motor impairments at the tested age.

In order to demonstrate that caspase-6 activation has a detrimental role on the severity of AD, we have established a caspase-6 knock-out (KO) colony and cross bred it with 5xFAD AD mice. These served as a new strain of AD mice which were tested in a variety of behavioral, histological, and biochemical exams, in order to evaluate the impact of caspase-6 KO in transgenic AD mice. Most studies using animal models today usually focus on either male or female mice in order to minimize discrepancies between treatment groups. However, in mice and specifically in transgenic AD models [44], gender can have a great impact on animal behavior, as well as developing pathologies [42]. This can be observed in cancer or pain studies [45,46,47] in mice, that show a complete change in the drug effect when testing male or female mice. Indeed, female mice, as well as humans, are known to be more prone to AD and other types of dementias [48,49]. The reason for this is not yet fully understood; some research points to menopause [50], the *BDNF* gene [51] or mitochondrial toxicity [48] amongst other possibilities. Another study has shown that female mice have higher caspase activation in a stroke model, and drug effects were vastly improved compared to male mice [52]. In our study, gender differences were apparent as well and can be attributed to variance in genetics, inflammatory response, and the mouse model used.

Memory impairments are apparent in the 5xFAD mouse model from an early age and can be evaluated using various behavioral tests. In the memory test phase of the Y-maze, female 5xFAD/C6-KO mice showed a significant improvement, compared to their transgenic counterpart, and spent more time in the novel introduced arm. In this test, male mice did not show an improvement over the 5xFAD/C6-Het group.

Reduced anxiety is a well-known characteristic of the 5xFAD model, depicted by a preference toward the open arm of the elevated plus maze [53]. This behavior usually manifests at six months of age [54], although other articles mention no significant difference between transgenic and WT male mice until nine months of age [20,55]. In the current study, male 5xFAD/C6-Het mice did not display the expected behavior, while the 5xFAD/C6-KO group spent 20% of their time in the open arms, exhibiting reduced anxiety. However, female 5xFAD/C6-KO mice showed a reduction in exploratory behavior closer to the WT groups in the elevated plus maze. In both the Y-maze and elevated plus maze, 5xFAD/C6-KO female mice performed significantly better than their transgenic counterpart. This result could indicate that caspase-6 KO has an effect on anxiety-like behaviors and short-term memory in female mice.

The hippocampus is one of the main areas of the brain affected by amyloid deposition and neuronal death in AD brains [56]. This is also the case in the transgenic mouse model [18] and can be applied to relevant behavior tests, such as the Morris water maze (MWM). Indeed, both genders of transgenic mice required several days to learn the platform position. In contrast, both male and female 5xFAD/C6-KO mice showed a rapid learning curve in the MWM, with a significant improvement on the second day of learning. Male KO mice fared better at finding the platform position, and by the fourth day of learning they surpassed their 5xFAD counterpart. The probe test also showed significant amelioration in the time spent searching inside the platform zone in both male and female mice. This test shows amelioration in hippocampal working memory in 5xFAD/C6-KO mice, one of the most affected areas of the brain in AD, and associates caspase-6 activity as a negative effector in the disease.

Previous studies have shown that caspase-6 could mediate synapse dysfunction and loss before the occurrence of neurodegeneration and cell death [22]. Another study used a knock-in mouse that expresses a self-activated form of human caspase-6 in the CA1 and has shown that synaptophysin was reduced when caspase-6 is overexpressed [38]. Therefore, in our study, we wanted to see if knocking out caspase-6 has a beneficial effect on synapse dysfunction. Analyses of male and female hippocampi revealed a drastic increase in synaptophysin in 5xFAD/C6-KO mice, a protein constituent of synaptic vesicles in neurons, thus suggesting increased synaptic densities. Increase in synaptophysin expression is correlated with long-term potentiation and may contribute to learning and memory [57,58].

Inflammation is an integral part of multiple brain diseases [59] and is thought to be a central mechanism in AD [60]. Therefore, we evaluated mRNA levels of the pro-inflammatory cytokine TNF-α in mice hippocampi and found it to be significantly reduced in 5xFAD/C6-KO male, but not female, mice compared to their transgenic counterpart. TNF-α is known to be in close relationship with caspase-6, with its expression increasing when caspase-6 is active [61].

An additional cytokine of anti-inflammatory nature, IL-10, is an important negative immune regulator that can counteract damage caused by excessive inflammation [25,62]. In both caspase-6 KO groups, IL-10 was found to be elevated compared to non-KO groups. This might suggest an anti-inflammatory process, originally hindered by caspase-6 activity, which could mediate reduced inflammation in KO mice.

In AD, additional pro-inflammatory cytokines are known to be up-regulated, such as IL-6 and IL-1β [63,64]. IL-6 can lead to induction of APP expression, but can also suppress Aβ deposition in vivo [65]. IL1-β increase in AD is believed to be correlated with memory impairments [66], and to promote neuronal and synaptic dysfunction [67]. On the other hand, IL-1β elevation may induce plaque degradation by increasing microglial activation and phagocytic activity [68]. Gene expression analysis of IL-6 and IL1-β genes in both 5xFAD/C6-KO and 5xFAD/C6-Het mice were significantly elevated compared to that of their WT counterparts. These cytokines can be up-regulated by different pathways, without the requirement of caspase-6 [69] and are at times referred to as beneficial in neuroprotection through inflammatory pathways [64,70].

Amyloid-β plaque deposition, albeit slight controversy, is still to this day considered the main pathology of AD [3,71,72,73]. Reduction in Aβ plaques in transgenic mouse models were regarded as an endpoint for the studies of AD [74,75]. Caspase-6 has been implicated in aiding in the generation of Aβ plaques [7,76,77] and propagation of the APP cleavage [52]. In this experiment, a significant reduction in Aβ aggregate staining was observed in both male and female double transgenic 5xFAD/C6-KO mice, which further demonstrates the link between Aβ and caspase-6 activity in AD. Reduction in amyloid plaque burden in 5xFAD/C6-KO mice can further attest for the improvement in animal spatial memory and learning behavior, as amyloid-β is in direct correlation with hippocampal memory formation [33,34].

Microglia, resident macrophages of the neurological system, act as a defense mechanism for the central nervous system. Together with astrocytes, they create a mesh of activity that leads to inflammation and the progression of AD [78,79]. In our study, we have shown a reduction in both these neurological hallmarks through immunohistochemistry, which leads us to believe that caspase-6 has a direct effect on microglia and astrocyte activation in this mouse model of AD. Signaling from microglia through pro-inflammatory cytokines, such as TNF-α, can lead to the activation of apoptosis in neurons through caspase-dependent pathways [80]. In the 5xFAD/C6-KO mice, we see a decrease in both microglia activation, as well as *TNF-α* gene expression. Combined with the reduction in pro-apoptotic caspase-3 levels in the hippocampus, we observe a series of reactions that can protect the 5xFAD/C6-KO mice from further neuronal death.

Our study showed that caspase-6 KO has beneficial effects in a mouse model of severe AD, and therefore demonstrates further proof that it plays a fundamental role in the progression and severity of the disease. Several key behaviors were improved, and protein expressions were affected towards the betterment of AD outcome. We believe this is additional confirmation that caspase-6 is associated with the pathogenesis of AD at varying stages, and that caspase-6 inhibition can lead to neuronal protection.

Further studies should be performed to expand on caspase-6 inhibition, and to use it as a new target for prevention.

## 4. Materials and Methods

### 4.1. Animals

All rodent procedures were approved by the Tel Aviv University Institutional Animal Care and Use Committee (Ref No. 01-17-071, approved 06 September 2017). Mice were maintained in 12-h-light/12-h-dark conditions in individually ventilated cages with ad libitum access to food and water. Every effort was made to reduce the number of mice used and minimize their suffering.

### 4.2. 5xFAD Mouse Model

The 5xFAD mouse model has three familial AD mutations in the amyloid precursor protein (APP) transgene [K670N/M671L(Swedish)+I716V (Florida)+V717I (London)] and two mutations in the presenilin-1 (PSEN1) transgene (M146L+L286V) [18]. Hemizygous transgenic 5xFAD males (JAX #006554) were a generous gift from Professor Dan Frenkel, and WT females (C57BL/6J) were obtained from Envigo (Rehovot, Israel) as breeding pairs. We bred 5xFAD hemizygous males with C57BL/6J females. Given that APP and PS1 transgenes co-segregate in 5xFAD mice, offspring were either hemizygous or WT. Mice were genotyped for both the APP and PS1 transgenes with polymerase chain reaction (PCR) using tissue samples obtained via tail piece (AccuStart II Mouse Genotyping Kit - Quanta Bio, Beverly, MA, USA).

### 4.3. Caspase-6 Knock Out Mice

Caspase-6 null (Caspase-6 +/-) mice [19] (Jackson Laboratories, #006236) on a C57BL/6 background were bred amongst themselves to generate Caspase-6 knock-out (-/-), which was confirmed through tail PCR. Genotyping was performed using the forward and reverse primers provided by Jackson (IMR5940, IMR5941, IMR5942) and following their protocol for Caspase-6 KO mice.

### 4.4. Combination of 5xFAD with Caspase-6 KO

Homozygote males for Caspase-6 KO were bred with 5xFAD female mice to generate Caspase-6 (-/-) and 5xFAD hybrids, as well as wild-type littermates. Animals were stratified to cages at one month of age according to their genotype and gender. The final genotypes used in the study are depicted in supplementary Table S1. Mice were handled periodically, and behavioral testing started at six and a half months of age.

### 4.5. Behavioral Tests

#### 4.5.1. Elevated Plus Maze

The elevated plus maze is generally used for the assessment of anxiety-related behavior. A plus-shaped maze containing two dark and enclosed arms, and two open and lit arms elevated 100 cm above ground, was used. The arms were 30 × 5 cm with a 5 × 5 cm center area, and the walls of the closed arms were 40 cm high. Mice were placed in the center of the maze, tracked for 5 min with a video camera, and then returned to their home cage. Time spent in the open arms were measured using Ethovision (v11.5, Noldus, Wageningen, Netherlands) video tracking system.

#### 4.5.2. Y-maze

Forced alternation Y-maze was performed to assess spatial memory, as previously described [81,82]. The test was conducted in a white, Perspex Y-shape apparatus with arm length of 38 cm, width of 5 cm, and height of 15 cm. The test consisted of a sample trial and a test trial. In the sample trial, the mice were placed at the end of one arm of the maze facing the wall, while one arm of the maze was blocked, and mice could explore the two arms of the maze for 5 min. The sample trial was followed by a 5 min inter-trial interval. In the test trial, the mice were returned to the maze with all arms open for another 5 min. Novel arm exploration time was measured for the duration of the test trial.

#### 4.5.3. Morris Water Maze

Mice were assessed for memory retention and cognition in the Morris water maze (MWM) at seven months of age. The test consisted of a large pool of water with visual cues and a hidden platform located in the same quadrant throughout the learning phase. Mice were released from a different quadrant in the pool four times per day for 60 s trials during the four-day learning period. During the learning phase, mice that did not find the platform were encouraged towards the platform and left untouched for 30 s. Latency to reach the platform was calculated each day as a mean of all trials. Mice that failed to find the platform were scored as having reached the platform in 60 s. On the fifth day, the platform was removed, and mice were released from the opposite side for a 60 s probe trial. Time spent in the platform quadrant was tracked using Ethovision 11.5 software.

### 4.6. Real Time-PCR

Hippocampal RNA was extracted using RNeasy Mini Kit (Qiagen, Hilden, Germany), as previously described [83]. RNA was reverse transcribed to complementary DNA (cDNA) using verso cDNA synthesis kit (Thermo Fisher Scientific, Waltham, MA, USA). Semi-quantitative PCR was performed on the Step-One Real time PCR (RT-PCR) system using Syber-Green Master mix (Thermo Fisher Scientific) and custom designed primers. Threshold cycle values were determined in triplicates and presented as average, compared with actin. Fold changes were calculated using the ^2∆CT^ method.

#### Primer List (Mouse Genes)

TNFα Forward: 5′-AGGGTCTGGGCCATAGAACT-3′ and Reverse: 5′-CCACCACGCTCTTCTGTCTAC-3′; IL-10 Forward: 5′-GAGAGCTGCAGGGCCCTTTGC-3′ and Reverse: 5′-CTCCCTGGTTTCTCTTCCCAAGACC-3′; Caspase-3 Forward: 5′-TGACTGGAAAGCCGAAACTC-3′ and Reverse: 5′-AGCCTCCACCGGTATCTTCT-3′.

### 4.7. Histology

#### 4.7.1. Sample Preparation

At eight months of age, most animals were euthanized with CO_2_, and the brains were immediately removed. Hippocampi, pre-frontal cortex, and cerebellum were dissected and snap-frozen in liquid nitrogen immediately. Tissues were kept at −80 °C until use. These samples were used for western blot analysis and RT-PCR.

#### 4.7.2. Immunohistochemistry

At eight months of age, the remaining animals were anesthetized with ketamine/xylazine (MediMarket, Emek Hefer Israel/ Eurovet, Bladel, Netherlands) and transcardially perfused with cold phosphate buffer saline (PBS), followed by 4% paraformaldehyde (PFA, #158127, Sigma-Aldrich, Jerusalem, Israel). The brains were then fixed with 4% PFA overnight, equilibrated in 30% sucrose for 48 h, then transferred to PBS Azide (0.02%) solution for preservation until cryo-sectioning. Brains were sectioned (10 μm) using a cryostat and mounted directly onto slides for analysis.

For immunohistochemistry, slides were incubated with blocking solution (5% goat serum, 1% BSA, 0.05% Triton-X in PBS) for 1 h at room temperature (RT), then incubated overnight at 4°C with the following primary antibodies: rabbit anti-GFAP (1:500, ab7260, Abcam, Cambridge, United Kingdom), rabbit anti-IBA1 (1:500, ab178847, Abcam). Then, sections were incubated with secondary antibodies: goat anti-rabbit Alexa 568 (1:700, Invitrogen, Carlsbad, California, USA) for 1 h at RT. The nuclei were stained with DAPI (1:1000, Sigma-Aldrich). For microscopic analysis, a Leica SP5 confocal laser scanning microscope was used (Leica microsystems, Wetzlar, Germany). Intensity of fluorescence was measured using ImageJ software (ImageJ software v1.6.0, NIH, Bethesda, MA, USA). At least three brains for each group were used for quantification. Results represent the averages of each group.

For Thioflavin S (ThioS, T1892, Sigma-Aldrich) staining, following the blocking step, slides were incubated for 8 min with 0.01% ThioS solution in 50% ethanol. Slides were then briefly incubated twice for 10 s with 80% ethanol and washed twice with double distilled water (DDW).

### 4.8. Protein Extraction and Analysis

Tissues were thawed, and homogenized in lysis buffer (200 mM HEPES, 5 mM EDTA, 1% Nonidet P-40, 0.5% sodium deoxycholate, 1 mM Na2VO4, 150 mM NaCl, and 50 mM NaF) supplemented with protease inhibitor (Roche, Basel, Switzerland). Lysates were incubated for 1 h at 4°C. Proteins were cleared by centrifugation at 14,000 RPM for 20 min at 4°C. Protein concentrations were quantified utilizing the Pierce^TM^ BCA Protein Assay Kit (Thermo Fisher Scientific). Thirty micrograms of protein from each sample was resolved in SDS-PAGE. Nitrocellulose transferred membranes were blocked for 1 h with PBS 0.1% Tween-20 with 5% bovine serum albumin (BSA), probed with goat anti synaptophysin antibody (1:1,000, sc-9116, Santa Cruz, Dallas, TX, United States) overnight at 4°C, and with goat anti actin antibody (1:5000, MAB1501, Milipore, Burlington, MA, United States) for 1 h at RT, followed by incubation with goat anti Rabbit secondary antibody (1:10,000, Licor, Lincoln, NE, United States) for 1 h at RT. Visualization and analysis of band intensities were performed using the Odyssey system (Licor) and the Image Studio Lite 5.2 software. For each sample, primary antibody results were normalized to actin.

### 4.9. Statistical Analysis

The results are expressed as means ± standard error mean (SEM). Statistical analysis was performed using unpaired Student’s t test for the direct comparison between two groups. Statistical analysis of data sets was carried out with the aid of GraphPad Prism 6.01 for Windows (Graphpad Software, CA, USA). All authors have read and agreed to the published version of the manuscript.

## Supplementary Materials

The following are available online at https://www.mdpi.com/1422-0067/21/3/1144/s1.

## Abbreviations

Aβ	Amyloid-β
AD	Alzheimer’s disease
APP	Amyloid precursor protein
C6	Caspase-6
cDNA	Complementary DNA
DAPI	4,6-diamino-2-phenylindole
FAD	Familial Alzheimer’s disease
GFAP	Glial fibrillary acidic protein
Het	Heterozygous
KO	Knock-out
IHC	Immunohistochemistry
Mg	Milligram
MWM	Morris water maze
NS	Not statistically significant
PBS	Phosphate buffer saline
PCR	Polymerase chain reaction
PFA	Paraformaldehyde
Q1	Quadrant #1
RNA	Ribonucleic acid
RT	Room temperature
RT-PCR	Real time polymerase chain reaction
SEM	Standard error of the mean
ThioS	Thioflavin-S
