# Supplementary material for: Caspase-6 Knockout in the 5xFAD Model of Alzheimer’s Disease Reveals Favorable Outcome on Memory and Neurological Hallmarks
Source: Int J Mol Sci. 2020 Feb 9;21(3):1144. doi: 10.3390/ijms21031144 (PMC7037950; doi:10.3390/ijms21031144)
Supplement: Supplementary file 1 [file ijms-21-01144-s001.pdf]

Caspase-6 knockout in the 5xFAD model of Alzheimer's disease reveals favorable outcome on memory and neurological hallmarks

Ariel Angel[1], Rotem Volkman[1], Tabitha Grace Royal[3], Daniel Offen[1,2]

Supplementary material:

Supplementary Table S1 - Genotype summary and group stratification

Genotype	Description	Abbreviation	Number of animals	
			Female	Male
5xFAD (-/-) x Caspase-6 (+/-)	Wild type x Heterozygote	WT/C6-Het	11	9
5xFAD (-/-) x Caspase-6 (-/-)	Wild type x KO	WT/C6-KO	13	12
5xFAD (+/-) x Caspase-6 (+/-)	Transgenic x Heterozygote	5xFAD/C6-Het	17	15
5xFAD (+/-) x Caspase-6 (-/-)	Transgenic x KO	5xFAD/C6-KO	18	12

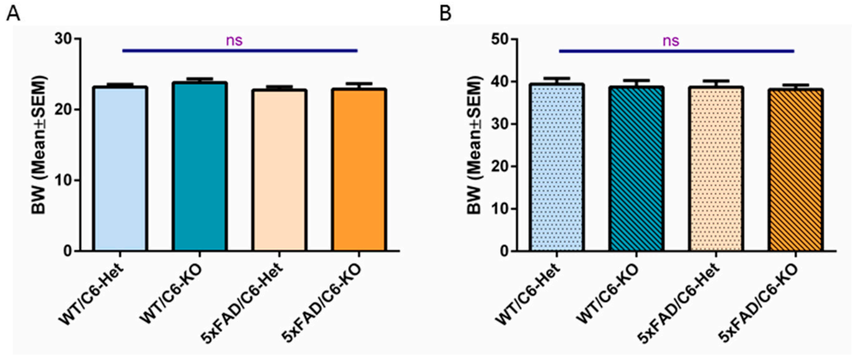

Figure S1- Average body weight of animals was identical throughout each group in accordance with gender.

Average body weight (BW) at 7 months for female (A) and male (B) mice

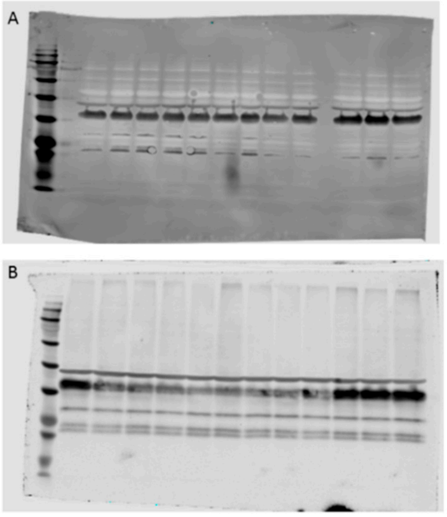

Figure S2 – Uncropped western blots for synaptophysin

Male (A) and female (B) immunoblots for synaptophysin and actin. Legends available on Figure 4.

Caspase-6 knockout in the 5xFAD model of Alzheimer's disease reveals favorable outcome on memory and neurological hallmarks

Ariel Angel[1], Rotem Volkman[1], Tabitha Grace Royal[3], Daniel Offen[1,2]

Supplementary Table S2 – Gender differences in 5xFAD/C6-KO mice

Parameter evaluated	Result in females 5xFAD/C6-KO	Result in males 5xFAD/C6-KO
Y-Maze	Significant increase in time spent in novel arm	No effect compared to 5xFAD/C6-Het
Elevated plus maze	Significant decrease in time spent in open arms	No model established
Morris water maze - Learning	Faster learning on the second day. No further improvement in subsequent days	Faster learning from the second day. Significant improvement by the fourth day
Morris water maze – Probe test	Slight increase in time spent in quadrant	Significant increase in time spent in quadrant
Synaptophysin	Significant increase in protein expression in Hippocampus	Significant increase in protein expression in Hippocampus
TNFα mRNA	No effect compared to 5xFAD/C6-Het	Significant decrease compared to 5xFAD/C6-Het
IL-10 mRNA	Significant increase compared to 5xFAD/C6-Het	Significant increase compared to 5xFAD/C6-Het
Caspase-3 mRNA	No effect compared to 5xFAD/C6-Het	Significant decrease compared to 5xFAD/C6-Het
Amyloid plaque	Significant decrease in plaque formation in Hippocampus	Significant decrease in plaque formation in Hippocampus
GFAP	Significant decrease compared to 5xFAD/C6-Het	No effect compared to 5xFAD/C6-Het
IBA1	Significant decrease compared to 5xFAD/C6-Het	Significant decrease compared to 5xFAD/C6-Het

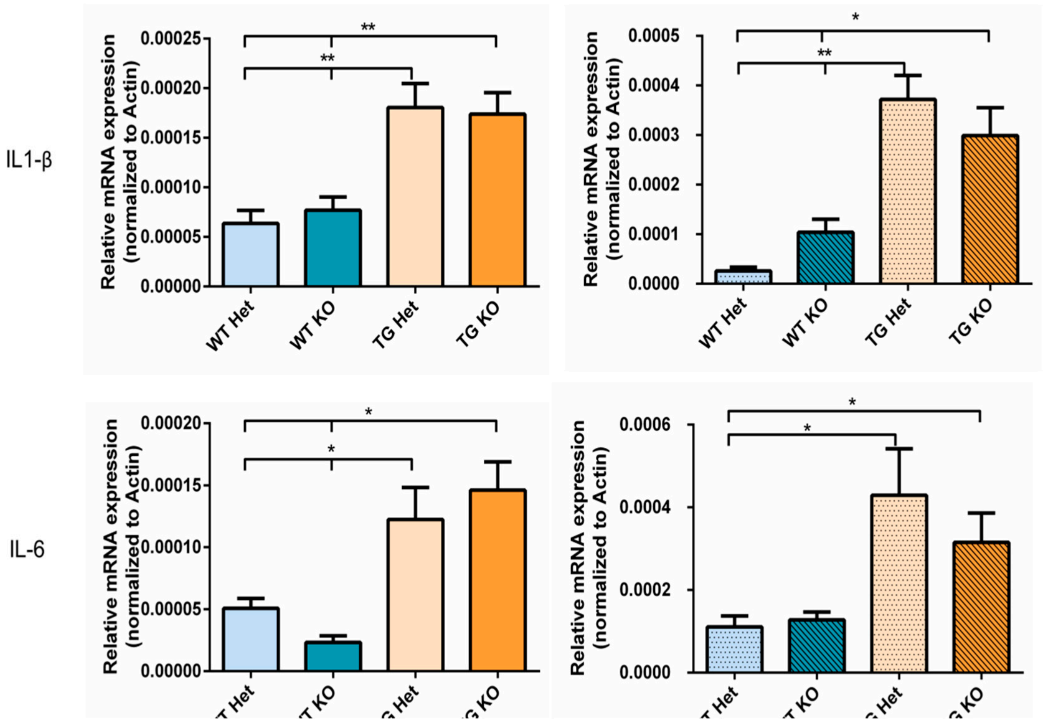

Supplement Figure S3 – Pro-inflammatory cytokines gene expression in mice hippocampi

mRNA quantification of pro-inflammatory cytokines IL1-β and IL-6 from female and male mice hippocampi. No significant difference was found between the two AD groups. Data are mean ± SEM. *P < 0.05, **P < 0.01. Two-tailed t-test between marked groups.
